# Caffeine, MitoQ, and GABA Prophylaxis of Mitochondrial Dysfunction Induced in Human Pulmonary Cells by Normobaric–Hyperoxia and Hyperbaric–Hyperoxia

**DOI:** 10.1155/omcl/5589475

**Published:** 2025-09-22

**Authors:** Tanvir Hossain, Jackson T. Secor, David M. Eckmann

**Affiliations:** ^1^Department of Anesthesiology, The Ohio State University, Columbus 43210, Ohio, USA; ^2^Center for Medical and Engineering Innovation, The Ohio State University, Columbus 43210, Ohio, USA

**Keywords:** hyperbaric, mitochondria, oxygen, pulmonary, toxicity

## Abstract

Exposure to hyperoxia lasting either a few days at normobaria or a few hours at hyperbaria induces pulmonary oxygen toxicity. Cellular functional changes resulting from oxygen toxicity include alterations in both mitochondrial dynamics and bioenergetics. The primary goal of this study was to quantify the prophylactic effects of three compounds, caffeine, MitoQ, and γ-aminobutyric acid (GABA), to protect human pulmonary cells in vitro from mitochondrial alterations induced by normobaric- and hyperbaric–hyperoxic conditions. Using cultured lung microvascular and pulmonary artery endothelial cells as well as A549 cells, we examined mitochondrial dynamic and bioenergetics function following exposure to normobaric–hyperoxic (5% CO_2_ and 95% O_2_ for 72 h) and hyperbaric–hyperoxic (~5% CO_2_ equivalent and remainder O_2_ at pressure of 4.8 atmosphere absolute (ATA) for 4 h) conditions in the presence of the drugs. Mitochondrial respiration parameters, inner membrane potential, motility, intracellular distribution, and size were measured, along with quantitation of respiration complex levels. Redistribution of intracellular ATP-linked respiration was determined. Comparisons of results were made to controls under normobaric–normoxic conditions. Effects of the drugs under control conditions were also measured. Presence of the drugs resulted in differential effects on hyperoxia-induced alterations in cellular respiration function, stability of mitochondrial potential, and distribution of ATP-linked respiration within the cell. Inclusion of these drugs also produced unique signatures for respiration complex protein levels. Moreso for caffeine than for MitoQ and GABA, its inclusion in the face of hyperoxic exposure served to preserve mitochondrial bioenergetics function, primarily by promoting intracellular redistribution of mitochondrial volume to the perinuclear space. These results indicate a potential role for pharmacologic prophylaxis via therapeutics targeted to support mitochondrial function as a means of protecting the lung from hyperoxia-induced pulmonary cellular oxygen toxicity.

## 1. Introduction

The pulmonary system is highly disposed to development of oxygen toxicity when exposed to oxygen at high partial pressures. The lung's exquisite sensitivity to oxygen occurs in part because it is comprised of over 40 distinct cell types [1]. Two particular human populations are subject to high concentration oxygen exposure that increases the likelihood that pulmonary oxygen toxicity will occur. First are ill individuals in intensive care unit (ICU) settings, where intubated patients are frequently ventilated mechanically using oxygen at inspired levels of 0.6–1.0 atmosphere absolute (ATA), also referred to as normobaric conditions [2–8]. Pulmonary oxygen toxicity associated with normobaric exposures occurs over two to 3 days and is marked by diffuse damage evident in pulmonary capillary endothelial cells as well as alveolar epithelial cells. The related pathophysiology involves inflammation with inflammatory cell infiltration, intra-alveolar and interstitial edema, and significant decrements in gas exchange [9–12]. Respiratory failure and death are known ensue beginning as early as 72 h with continuous administration of 100% oxygen (i.e., 1 ATA) [13, 14].

The second at-risk population is comprised of individuals breathing hyperbaric oxygen (HBO) at 2 ATA or higher. This includes SCUBA divers and ill patients who are receiving HBO therapy for a variety of clinical indications. HBO-associated pulmonary oxygen toxicity develops rapidly following even just a few hours of hyperbaric exposure [5, 6]. While characteristically it resembles normobaric pulmonary oxygen toxicity in the development of microscopic and gross pathological tissue changes, hyperbaric pulmonary oxygen toxicity develops much faster and exhibits less direct inflammatory-related injury and more noninflammatory-related injury than occurs in its normobaric counterpart [15].

The severity and lethality of oxygen-induced lung toxicity renders in vivo research models highly challenging. Much of the knowledge regarding pathophysiologic and toxicokinetic causes of, and potential treatments for, oxygen toxicity has come from cellular models. Cell-based studies have demonstrated that metabolic derangements based in mitochondrial dysfunction readily occur [16]. Subcellular and molecular events are central to the pathophysiology of oxygen toxicity, as investigations of lung injury resulting from normobaric and HBO have proven [17–20]. Our recent work involving multiple pulmonary cell lines in vitro has clearly demonstrated that both normobaric and HBO exposure impair the production of ATP by mitochondria, especially negatively impacting the available supply of ATP for the cell nucleus [3, 21, 22]. Hyperoxia also alters mitochondrial motility, effects the intracellular distribution of mitochondria, disturbs maintenance of the inner membrane potential, and influences the level of expression of proteins constituting the electron transport chain (ETC) producing ATP [3, 22]. Our studies also show that the mitochondrial bioenergetic and dynamic signatures resulting from normobaric and HBO exposures are not identical [3, 22]. These findings further bolster the conclusion put forward in [15] from animal-based work that normobaric and HBO toxicity are similar, but not the same.

Impairment of mitochondrial oxygen utilization with resultant loss of ATP production can give rise to lung pathophysiology at multiple scales including molecular (e.g., ETC proteins), organelle (e.g., mitochondria), cellular (e.g., pulmonary capillary endothelium), and entire organ (e.g., pulmonary edema and impaired gas exchange) [23, 24]. These manifestations of pulmonary oxygen toxicity drive the clinical requirement for intensive care and are the root cause of patient deaths. There exist only limited clinical interventional options to prevent or treat pulmonary oxygen toxicity. Prevention is focused on limiting use of high concentrations of oxygen, but this is not always achievable. The only current treatment strategy for oxygen toxicity is to decrease the fraction of inspired oxygen to the lowest concentration tolerable to minimize tissue hypoxia.

The work reported here was implemented to study the effects of three different compounds, namely caffeine, γ-aminobutyric acid (GABA), and MitoQ to protect three distinct pulmonary cell lines (human pulmonary artery endothelial cells [HPAECs], A549 cells, and human lung microvascular endothelial cells [HLMVECs]) cultured in vitro from the mitochondrial bioenergetic and dynamic dysfunction previously shown to be induced by normobaric- and hyperbaric–hyperoxic conditions [22]. These lung cell types represent cells present in the airway and the lung vasculature that may be susceptible to oxygen toxicity. They are involved in regulation of nutrient transport, signaling, and gas exchange functions constituting routine pulmonary physiology [25–28]. The A549 human lung adenocarcinoma cell line is well established as a type II pulmonary epithelial cell model [29]. Injury to these cell lines resulting from hyperoxia may produce adverse lung physiology that could precipitate respiratory failure or even death. The two environmental conditions imposed on the cells have previously been demonstrated to produce quantifiable decrements in mitochondrial function [3, 22]. These two distinct hyperoxia exposures were selected in recognition of the fact that normobaric and HBO toxicity are only similar, giving credence to the notion that a therapeutic might be effective for one form, but not the other, of pulmonary oxygen toxicity. We systematically evaluated whether any protective effects from oxygen toxicity were agent-specific, cell-type dependent, or generalizable across the two models that produce oxygen toxicity. This framework enables distinctions to be made between shared versus unique pharmacological protections across multiple stress models.

## 2. Materials and Methods

### 2.1. Details of the Experimental Model

#### 2.1.1. Cell Culturing

Three cell line were used throughout the experiments. A549 cells (catalog number CCL-185, ATCC) were cultured in Ham's F-12 Kaign's medium (catalog number 21127-022, ATCC) with 10% fetal bovine serum (FBS) (Gibco catalog number A5256701). The HPAECs (catalog number CC-2530, Lonza), and HLMVECs (Lonza catalog number CC-2527) were cultured in endothelial cell basal medium-2 (EBM-2) (catalog number 3156) and supplemented with microvascular endothelial cell growth (EGM-2 MV) BulletKit (Lonza catalog number CC-4147). A549 and HPAECs were used during passage numbers 3–7, whereas HLMVECs were used during passage numbers 5–7. Cells were cultured in T25 flasks (product ID:10062-872, VWR) until an 80%–90% confluency was achieved. For imaging, we plated ~4000 cells onto 2 mL microscopy dishes (catalog number 81156, Ibidi) 24 h prior to experimentation. For seahorse experiments, 60,000 cells were plated in each well of a 24-well seahorse XF cell culture plate that was previously coated with Cell-Tak and Tissue Adhesive (Corning catalog number 354240) to ensure that cells adhered to the plate, as described in detail in prior literature [3, 17, 20–22, 30]. All cells were maintained in an incubated environment (catalog number MCO-170AICUV-PA, Panasonic) at 37°C with 5% CO_2_.

#### 2.1.2. Drug Concentration Determination and Utilization

For all three drugs tested in this model, a brief analysis of pertinent research articles was first undertaken to determine a suitable dosage range to be applied initially in our experiments. Once a range was identified from literature, the range was then evaluated through detailed respiration analysis using the three cell lines at standard culture conditions in preliminary experimentation in order to identify a single concentration of each drug to be used in the subsequent full set of experiments. The goal was to identify a single dose of each drug that demonstrated at most only modest effects, positive or negative, on baseline and/or maximal respiration levels without causing any signs of large-scale negative effects on these parameters.

For caffeine, which causes much of its biological effect through antagonizing adenosine receptors at concentrations below 250 μM [31], and which has been shown to potentiate the education of macrophages in culture conditions toward an anti-inflammatory phenotype at concentrations up to 1 mM [32], we examined its effects on respiration at 1, 5, and 10 mM. Through this analysis, we identified a caffeine concentration of 5 mM as a concentration having modest respiration effects without significant evidence of toxicity. For GABA, the three concentrations used for single dosing selection were 1, 5, and 10 μM. This range was based on GABA concentrations of 1–100 μM used in excised tissue studies of taste receptor cell signaling [33], a concentration of 50 μM studied in cultured cerebellar granule cells [34], and concentrations ranging from 0.14 μM to 33 μM examined in receptor binding affinity studies using cultured human neurons derived from pluripotent stem cells [35]. Our preliminary experiments yielded a GABA concentration of 1 μM for inducing modest respiration responses without serious toxicity being evident. For MitoQ, the three concentrations used for dose selection were 50, 100, and 200 nM. This range was based on concentrations of 100–500 nM studied in vitro in multiple cancer cell lines [36], effects of 50, 100, and 150 nM MitoQ on cultured ovarian organoids [37], and effects of 50, 100, 200, and 400 nM MitoQ on oxidative stress in stored yak semen [38]. Based on our preliminary experiments, 50 nM MitoQ resulted in modest respiration effects absent any major toxic effects.

Each of the three drugs was added to fresh cell media such that its final concentration was at the target level. For cell loading with drug prior to an experiment, cells were first washed and fresh media containing a drug was added for a 30 min uptake period under incubator conditions. This is a sufficiently long time for cellular uptake of each of the three study drugs [39–41]. Cells were then removed from the incubator, quickly washed, and fresh drug-free media was added. Cells were immediately placed into the experimental environmental exposure (normobaric- or hyperbaric–hyperoxic conditions) and then subjected to mitochondrial bioenergetics and dynamics assessment. With normobaric–normoxic conditions, cells were immediately subjected to mitochondrial bioenergetics and dynamics assessment following the drug incubation process.

### 2.2. Method Details

#### 2.2.1. Normobaric–Normoxic Exposure Conditions

Each of the baseline (control) experiments was conducted at local atmospheric pressure using a gaseous environment containing 20.9% O_2_, 5% CO_2_, and 74.1% N_2_. This same pressure and gas mix condition was used with each of the cell lines with each of the study drugs included.

#### 2.2.2. Normobaric–Hyperoxic Exposure Conditions

As performed previously [3], cell culture dishes were transferred to a sealed Billups-Rothenberg modular incubator chamber (Pat. Number 5352414). The chamber was flushed throughout for 5 min with a mixture of 95% O_2_ and 5% CO_2_ to drive out ambient gasses and fill the chamber with the desired hyperoxic mixture. Once flushed, the modular incubator was sealed to ensure the hyperoxic mixture was maintained in the container at ambient atmospheric pressure. The modular incubator was then stored into a large Panasonic (MCO 170AICUV) incubator at 37°C for 72 h. This same pressure and gas mix condition was used with each of the cell lines with each of the study drugs included.

#### 2.2.3. Hyperbaric–Hyperoxic Exposure Conditions

As described in [21, 22], cell culture dishes were transferred to a hyperbaric chamber (Nat'L BD Model 1080) for hyperbaric–hyperoxic exposure. The chamber has an interior volume of 150 L. We inserted two 5-gallon sealed carboys full of water to reduce the gas volume needed during experimentation. The carboys also served a thermal reservoir, enabling maintenance of a stable temperature profile during the experimental timeframe. The chamber has four window ports for viewing the interior domain, and it is equipped with both fine and coarse valves to control either gas inflow or outflow in order to maintain a fixed interior pressure during conduct of an experiment.

Using a gas mixer (Witt model: KM 100-3ME) connected to medical grade gas cylinders, we filled the chamber with a gas mixture consisting of ~1% CO_2_ and balance O_2_ to a pressure of 70.56 ± 2 PSI (4.8 ATA), for a total exposure time of 4 h. This approach yields an ambient-equivalent 5% CO_2_ environment under hyperbaric conditions and prevents acidification of the media while the cells are under pressure [22]. An integrated pressure sensor (Mensor model CPG2500, San Marcos, TX) was used for precise pressure measurement within the chamber. A gas sensor (Analox model Aspida, Huntington Beach, CA) was used to sample the O_2_ and CO_2_ levels within the chamber via a sampling tube teed into the chamber's exhaust tube. We manually controlled the interior temperature of the chamber by heating it with three custom-manufactured external adhesive heating pads (DBK Industrial). As was done previously [22], the internal temperature was first brought to 37°C at the location where the dish with cells would be placed before any cells were actually inside the chamber. A pair of thermometers were held inside the chamber such that their bulbs were located approximately 0.5 cm above the cells in order to assess the ambient temperature prior to, and during, the cell exposure to experimental conditions. The temperature registered on the thermometers was easily seen through the chamber windows. Temperature was kept within the narrow range of 36–37.5°C through manual on/off cycling of the heating pads. Prior to experimentation, the heating pads were turned on to preincubate (warm) the chamber. Cells were placed inside the chamber, which was then closed and flushed at high flowrate for 5 min through an open vent to exchange its gas content with the experimental mixture. The vent was then closed, the target pressure condition inside the chamber was achieved and manually fine-tuned as needed to keep the hyperbaric status stable.

Following exposure to any of the defined pressure and gas mix combinations indicated above, cells were immediately examined for respiration analyses, mitochondrial dynamics measurements, and mitochondrial respiration complex protein levels as described elsewhere in the Methods. Importantly, in previous work neither the normobaric–hyperoxic nor the hyperbaric–hyperoxic conditions used in these experiments was sufficiently harsh to cause cell death, but was severe enough to induce nonlethal oxygen toxicity in each of the cell lines [3, 22].

#### 2.2.4. Assessment of Mitochondrial Respiration

Following exposure to a gas pressure/gas mixture environmental exposure, cellular respiration was assessed using a Seahorse XFe24 Analyzer (Agilent, Santa Clara, CA). The T25 flasks were removed from the experimental environment, the cells were washed with PBS, and then cells were detached using 0.05% trypsin. Trypsin inhibitor was added after cell detachment occurred. Detached cells were counted with an automatic cell counter (Counter countess-II, Invitrogen) and then immediately transferred into the Seahorse microplates which were previously coated with CellTak coating and preconditioned in an incubator at 37°C without CO_2_ exposure [3, 17, 20–22, 30]. To promote cell adhesion the microplates were centrifuged for 1 min at 500 rpm using a small centrifuge (Sorvall, product ID: ST8R, Thermo Scientific). Analysis of mitochondrial respiration commenced in under 30 min from the time cells were removed from their environmental exposure in all experiments.

Seahorse runs were performed with 5 mM glucose, 2 mM GlutaMAX, and 1 mM sodium pyruvate added to XF DMEM assay medium at pH 7.4 and 37°C. Prior to analyzing, the Seahorse instrument was prewarmed to ensure cells were analyzed at 37°C. Reagents were added in specific calibration port to obtain specific respiratory states, including baseline respiration, proton leak, and maximum respiration. Following baseline respiration measurement, proton leak was assessed by application of oligomycin (1 μM), an inhibitor of ATP synthase (Complex V). Carbonyl cyanide-4 (trifluoromethoxy) phenylhydrazone (FCCP, 2 μM) was then applied to dissipate the mitochondrial membrane potential. This step enabled measurement of maximum respiration and determination of spare respiratory capacity (SRC) as the difference between maximum and baseline respiration. The last step was application of rotenone (0.5 μM) to impair mitochondrial respiration by inhibiting Complex I and thereby facilitate measurement of nonmitochondrial respiration. The ATP-linked respiration was also determined by subtracting the proton leak from the baseline respiration. Values of these respiration parameters were each reported as the average derived from three separate measurements obtained during a single stage of an experiment for a single well. For each Seahorse run, at least three wells were not seeded with cells in order to enable control background level measurements. The respiration data obtained were normalized using this metabolic activity; background readings for each plate were calculated by averaging the OCRs from certain background wells. Background values were subtracted from all succeeding readings. Background well readings that were extreme or erratic were excluded. All experiments were repeated three times, conducted on separate days, and each well was individually analyzed. The resultant experimental data are presented as the mean ± standard deviation (SD).

#### 2.2.5. Assessment of Mitochondrial Dynamics

Mitochondrial dynamics was determined by measuring the distribution of net mitochondrial movement, mitochondrial number, and mitochondrial dimensions. Wide-field fluorescence microscopy imaging and image analysis was performed as described in significant detail in previous publications [3, 17, 30, 42, 43], and in brief as follows. The technique uses fluorescently labeled mitochondria within cells, acquiring a temporal sequence of microscopy images which are later analyzed via software that outputs multiple mitochondrial dynamics measurements [42]. After an initial process that enhances image clarity, individual mitochondria are tracked over time for their intracellular position, locomotion, and dimensions. The intracellular space is partitioned by algorithm into two separate regions: a perinuclear region that constitutes a 3 μm space immediately proximal to the cell nucleus; and a peripheral region representing the remainder of the intracellular space [3, 17, 21, 22, 43]. We note that ATP molecules synthesized inside a cell are produced and delivered by mitochondria which reside close by. The cell nucleus contains no mitochondria, providing relevancy for the determination of mitochondrial dynamics as well as energetics in a perinuclear space from which the nucleus' ATP is supplied. The software provides mitochondrial dynamics in both these regions. Mitochondrial motility is reported as a distribution of displacements that follow a log-normal distribution.

Image acquisition was performed in all experiments with an Olympus IX-51 wide-field inverted epifluorescence microscope as in prior studies [3, 17, 20–22, 30, 42–46]. After environmental exposure but before any images were obtained, the cells were transfected with DAPI as well as with a concentration of 40 particles/cell of CellLight Mitochondria GFP, BacMam 2.0 (Life Technologies, Grand Island, NY, USA). Cells were kept at 37°C for 20 min in darkness in Recording HBSS (HBSS pH 7.4 with 1.3 mM CaCl_2_, 0.9 mM MgCl_2_, 2 mM glutamine, 0.1 g/L heparin, 5.6 mM glucose, and 1% FBS) to maintain osmotic equilibrium as well as for the transport of essential inorganic ions during the image acquisition stage of experiments. Imaging was conducted swiftly so that cells were out of incubator conditions for under 30 min. The microscopy imaging system included an oil immersion objective lens (Olympus 40x), a camera (Hamamatsu ORCA, 2048 × 2040 pixels), a light source (Lumencor LED), and software (MetaMorph v. 7.10.5) for calibrating the system and for obtaining images. Plates were initially scanned with a FITC filter to demonstrate that high-resolution images were being acquired and for selection of clearly defined individual mitochondria that would be used in image analysis. Data analysis relied on evaluation of 101 consecutive images acquired every 3 s in a 5 min epoch. This approach reduced or eliminated photobleaching effects.

ImageJ and MATLAB software, respectively, were used to preprocess and analyze the images [3, 17, 21, 22, 30, 42, 43, 46]. The mitochondrial dynamics parameters, including motility, in each intracellular region were determined [3, 17, 21, 22, 43]. We perform this regional analysis to quantify any distinctions in mitochondrial dynamic behaviors occurring within either of the two intracellular regions.

To facilitate the image analysis, DAPI images were used to locate the cell nucleus' edge, and thus determine the boundary of the small intracellular perinuclear space. Our methodology to accomplish this using ImageJ software has been described previously [17, 21, 43]. We then implemented advanced image analysis to determine mitochondrial dynamics by applying our MATLAB program to the preprocessed images obtained [3, 17, 21, 22, 42, 43, 46, 47]. Information about how to obtain these algorithms or other relevant software for mitochondrial dynamics measurement is available in [47]. For this analysis, we evaluated mitochondria in cells after each of the environmental exposures using these well-tested and validated imaging methods and other laboratory assessment techniques. Each of the experiments was performed three times on different days to minimize inconsistency in the results. Results from experiments for a unique grouping of gas/pressure exposure, cell type, and drug used were combined in the analysis. At least 10 cells studied in three specific experiments performed on separate days were included in the analysis for each group. We only imaged a few cells in any one experimental run so as not to prolong the total time between cell removal from the environmental exposure and completion of image acquisition.

#### 2.2.6. Mitochondrial Inner Membrane Potential Determination

Mitochondria were loaded with tetramethylrhodamine methyl ester (TMRM, product ID: T668, Invitrogen) in order to facilitate microscopy image analysis of inner membrane potential. Immediately following environmental exposure as in [3, 21, 22], cells were incubated with 20 nM TMRM and DAPI in working solution at 37°C for 30 min in dark conditions. Cells were rinsed twice and mounted in Recording HBSS for image acquisition. Following established methodology [3, 17, 21, 22] the inner membrane potential in each intracellular subregion was measured. Briefly, we used ImageJ software to merge the TMRM and DAPI images, after which the resultant merged images were partitioned into the same two intracellular spaces used in the mitochondrial motility assessment described above. We made at least 12 distinct measurements of fluorescence intensity within each intracellular region per cell, with at least eight cells being imaged for each environmental exposure condition. The average fluorescence intensity within each intracellular region was determined. Background fluorescence intensity measurements were obtained in close proximity to mitochondria present within each intracellular region. The inner membrane potential value is the difference between the average pixel intensity of mitochondria and the average background intensity determined from the multiple measurements, which were made on at least three distinct days.

#### 2.2.7. Intracellular Bioenergetic Distribution Determination

Applying our methodology and nomenclature as in [3, 17, 21, 22, 43], we determined the regional distribution of bioenergetic capacity. This parameter is derived using the respiration, mitochondrial size, number of mitochondria, and inner membrane potential data. It partitions the total ATP-linked respiration that has been measured into its two separate perinuclear and peripheral intracellular components. Because there does not exist a method for measuring these regional contributions independently, it is necessary to utilize a technique that subdivides overall ATP-linked respiration into these two contributions. We first determine the portion of the overall volume of mitochondria located in each intracellular subregion using acquired data for the number of mitochondria and their dimensions. We applied a linearized association between mitochondrial potential and cellular respiration, as has been detailed in other work [48] and applied in this manner [3, 17, 21, 22, 43], to the measured mitochondrial potentials acquired for each intracellular subregion. With the resultant mitochondrial volume fractions and respiration rates thus determined for each intracellular subregion, we constructed the fractional contributions of each subregion to total ATP-linked cellular respiration as described below.

Mathematically, BC_scaled_, the scaled total bioenergetic capacity is the sum of the scaled bioenergetic capacity present in the perinuclear and peripheral regions.

(1) $$\begin{array}{c} {\text{BC}}_{\text{scaled}}={\text{BC}}_{\text{scaled},\text{ perinuclear}}+{\text{BC}}_{\text{scaled},\text{ peripheral}}. \end{array}$$

The scaled bioenergetic capacity in the perinuclear region is given as follows:

(2) $$\begin{array}{c} {\text{BC}}_{\text{scaled},\text{ perinuclear}}=\frac{\Delta \Psi_{m,\text{perinuclear}}^{*L}\times V_{\text{perinuclear}}^{\text{mito}}\times {\text{Resp}}^{\text{ATP}}}{{\text{Resp}}_{\text{control}}^{\text{ATP}}\times \left[\left(\Delta \Psi_{m,\text{ perinuclear}}^{*L}\;\times V_{\text{perinuclear}}^{\text{mito}}\right)+\left(\Delta \Psi_{m,\text{ peripheral}}^{*L}\times V_{\text{peripheral}}^{\text{mito}}\right)\right]}, \end{array}$$

while in peripheral region it is given as follows:

(3) $$\begin{array}{c} {\text{BC}}_{\text{scaled},\text{ peripheral}}=\frac{\Delta \Psi_{m,\text{ peripheral}}^{*L}\times V_{\text{peripheral}}^{\text{mito}}\times {\text{Resp}}^{\text{ATP}}}{{\text{Resp}}_{\text{control}}^{\text{ATP}}\times \left[\left(\Delta \Psi_{m,\text{ perinuclear}}^{*L}\times V_{\text{perinuclear}}^{\text{mito}}\right)+\left(\Delta \Psi_{m,\text{ peripheral}}^{*L}\times V_{\text{peripheral}}^{\text{mito}}\right)\right]}. \end{array}$$

In Equations (1)–(3), ΔΨ_*m*,perinuclear_^*∗L*^ and ΔΨ_*m*, peripheral_^*∗L*^ are the linearized cellular respiration factors in the perinuclear and peripheral regions, respectively, following the scheme in [48] using the mitochondrial potentials measured for the experiment; *V*_perinuclear_^mito^ and *V*_peripheral_^mito^ are the volumes of mitochondria in the perinuclear and peripheral regions, respectively, based on the measured mitochondrial number and dimensions (length and width or diameter) in each region; Resp^ATP^ is the total ATP-linked respiration (baseline minus leak) measured for a particular experiment; and Resp_control_^ATP^ is the ATP-linked respiration under control (normobaric–normoxic) conditions. This approach enables determination of the two fractional intracellular contributions to overall respiration, and hence is labeled the regional distribution of total bioenergetic capacity.

### 2.3. Mitochondrial Respiration Complex Levels

#### 2.3.1. Extraction of Protein and Sample Preparation

The steps for protein extraction and sample preparation followed the method described previously [22]. First, RIPA extraction buffer (product ID: ab156034) with a protease inhibitor (product ID: 78425, ThermoScientific) included was pipetted into cell culture flasks for harvesting. Cells were then sonicated (ultrasonic sonicator, product ID: FB50, Fisher Scientific) three times at 20 kHz for 30 s. Samples were preserved on ice during sonication. Following homogenization, the samples underwent centrifugation (serial ID: B5070203, Labnet International Inc.) for 5 min at 13,000 rpm and 4°C to yield a separate supernatant and pellet. The pellet was discarded and the supernatant was extracted and stored at −80°C. For Western blotting, vials of supernatant were first thawed on ice. A 20 μL aliquot was then pipetted into each well of a 16.5% Mini-PROTEAN Tris-Tricine 10-well Gel (product ID: 4563063, Bio-Rad). Gels were run for an hour at 200 V an electrophoresis power supply (Bio-Rad PowerPac 300, product ID: 165-5050). Blots were transferred from the gel using a membrane roll (Immun-Blot LF PVDF, product ID: 1620264, Bio-Rad). We rocked the membrane lightly for an hour using a platform shaker (New Brunswick Innova 2000, product ID: M1190-0000) at room temperature with BSA blocking buffer added. We then discarded the blocking buffer solution and washed the membrane with PBST. Lastly, a primary antibody solution was added and the membrane was gently rocked overnight while being refrigerated at 4°C. Western blotting was done three times.

#### 2.3.2. Antibodies

The primary antibodies utilized were the same as those described and used in [22]: the oxidative phosphorylation (OXPHOS) human antibody cocktail (product ID: ab110411, Abcam) at 1:2000 dilution; the β-tubulin mouse monoclonal antibody (product ID: 926-42213, Li-COR); as well as the VDAC recombinant rabbit monoclonal antibody (product ID: MA5-33205, Invitrogen) at 1:1000 dilution. These antibodies underwent refrigerated (4°C) incubation overnight in PVDF membranes. Five distinct mouse mAbs are premixed and optimized in the OXPHOS cocktail, and they are directed against the following specific mitochondrial complex subunits: CI subunit NDUFB8 (ab110242); CII-30kDa SDHB (ab14714); CIII-Core protein 2 (ab14745); CIV subunit I (ab14705); and CV alpha subunit (ab14748). Membrane blocking was performed with 6% BSA in phosphate buffer saline and 0.5% Tween-20 (PBS-T, product ID: 1610780, Bio-Rad and product ID: 1706531, Bio-Rad) before secondary antibodies were added to the membranes. These included IRDye 680RD Donkey anti-mouse (product ID: 926-68072, Li-COR), IRDye 680RD Donkey anti-rabbit (product ID: 926-668073, Li-COR) and IRDye 800CW Goat anti-Rabbit IgG (product ID: 926-32211, Li-COR), all used under gentle agitation at 1:10,000 dilution and room temperature for 1 h. This was followed by five 3-minute washes in PBS-T at room temperature and subsequent blot visualization.

#### 2.3.3. Western Blot Densitometry

A Li-COR system and ImageJ were employed for quantitative Western blot analysis of protein band intensity. In brief, raw 16-bit TIF images were obtained from the blots using the Li-COR instrumentation, and images were then processed using ImageJ. Bands of interest were selected using the rectangle tool, and background intensity profiles were also obtained. Entire lane profiles were plotted with the background values having been subtracted. The raw densitometry values Complex 1–5 levels were calculated using the wand tool, and these results were then normalized for plotting and statistical analysis using the associated VDAC levels obtained under each experimental exposure.

### 2.4. Quantification and Statistical Evaluation

The data in each of the figures and tables appear as mean ± SD. Statistical analyses as well as data presentation in graphic forms were prepared using SigmaPlot 16.0 software (Inpixon). We tested all data for normality using the D'Agostino and Pearson omnibus normality test. ANOVA with repeated measures was used to distinguish differences between groups. Where needed, the Tukey Kramer *t*-test was used for post hoc pair-wise comparisons in order to correct for multiple comparisons in detecting differences between groups as well as respiration states. A *p*-value < 0.05 indicated statistical significance. Significant statistical results are also indicated in the figures and table.

## 3. Results

### 3.1. Drug Effects on Mitochondrial Motility With and Without Hyperoxic Exposures

We imaged cells after their exposure to each of the three different environmental conditions with and without the three drugs being present to determine their specific effects on mitochondrial motility. To be consistent with graphical representations of prior results [3, 17, 20–22, 30, 42–44], the measured motility values, i.e., the net distances traveled by mitochondria in the acquisition timeframe, were plotted on a log-normal scale for data acquired within both the peripheral and perinuclear intracellular regions.

**Figure 1:**
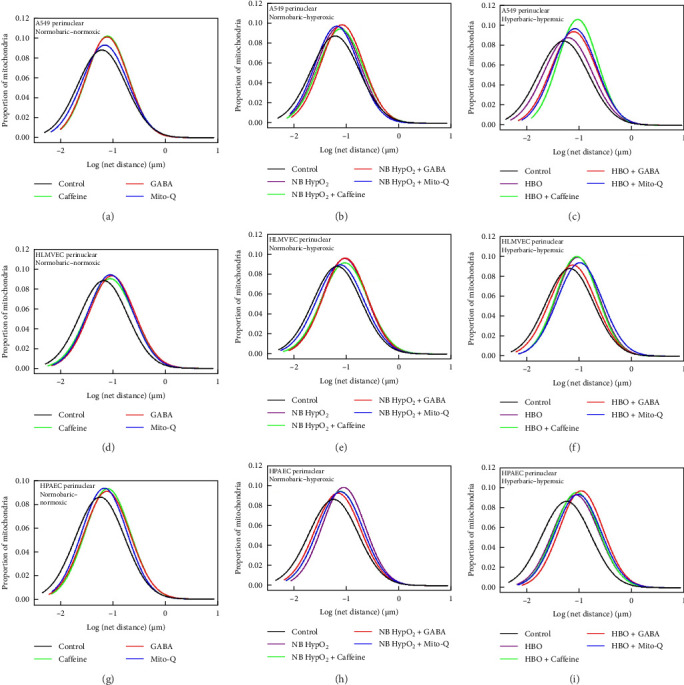
Perinuclear region mitochondrial motility in A549, HLMVEC, and HPAEC under different environmental conditions and with drugs present. Log-normal distribution of net distances traveled by mitochondria in the perinuclear region within A549 cells (a, b, c), HLMVECs (d, e, f), and HPAECs (g, h, i). Conditions studied were normobaric–normoxic (a, d, g), normobaric–hyperoxic (b, e, h), and hyperbaric–hyperoxic (c, f, i) exposures with and without caffeine, GABA, or MitoQ being present. The relevant curves for control values determined from normobaric–normoxic exposure and no drug present are also included in each panel for comparison.

**Figure 2:**
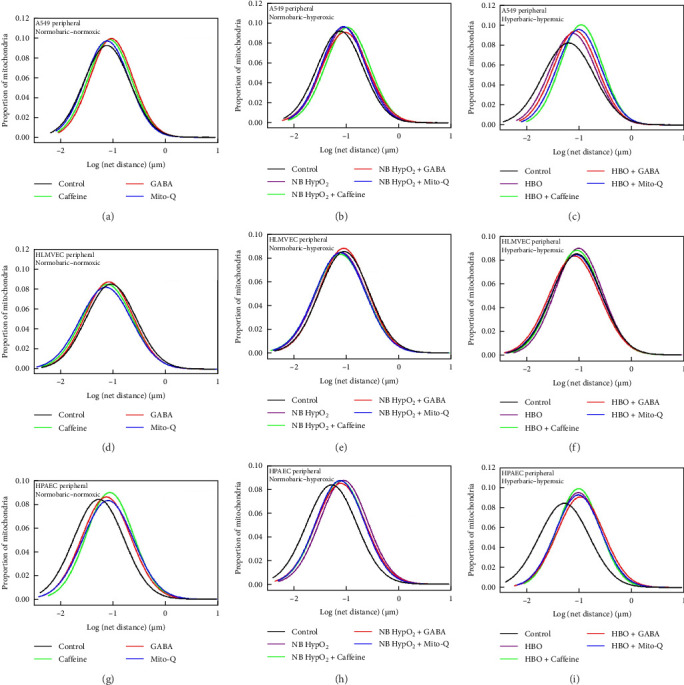
Peripheral region mitochondrial motility in A549, HLMVEC, and HPAEC under different environmental conditions and with drugs present. Log-normal distribution of net distances traveled by mitochondria in the peripheral region within A549 cells (a, b, c), HLMVECs (d, e, f), and HPAECs (g, h, i). Conditions studied were normobaric–normoxic (a, d, g), normobaric–hyperoxic (b, e, h), and hyperbaric–hyperoxic (c, f, i) exposures with and without caffeine, GABA, or MitoQ being present. The relevant curves for control values determined from normobaric–normoxic exposure and no drug present are also included in each panel for comparison.

Motility following normobaric–normoxic exposure in each type of the cell in both their perinuclear (Figure 1) and peripheral (Figure 2) regions was generally increased significantly from baseline by the presence of each of the three drugs. This is demonstrated by the rightward shift (i.e., increased motility) from the control curve present in the all three left-side panels in Figure 1 and in the left-side top and bottom panels in Figure 2. The exception to this generality was that motility was decreased significantly in the peripheral region of HLMVECs by the presence of each of the drugs under normobaric–normoxic conditions. This is shown by the leftward shift (i.e., decreased motility) from the control curve present in the left-sided middle panel in Figure 2. Further examination of the motility graphs in Figures 1 and 2 shows that addition of the drugs had differential effects on motility, i.e., either leading to an increase or a decrease in motility, depending on the particular cell line as well as the particular type of hyperoxic exposure. The graphs demonstrate that the measurements in these experiments recaptured the effects of both normobaric- and hyperbaric–hyperoxic on mitochondrial motility in the absence of the drugs as previously reported [3, 22]. Inclusion of the drugs yielded a further increase in motility with an additional rightward shift in some cases (e.g., effects of caffeine, GABA, and MitoQ in A549 cells exposed to hyperbaric–hyperoxic conditions, top-right panels in Figures 1 and 2). In other cases the drug inclusion resulted in a leftward shift in motility from the hyperoxic-exposure alone (no drug inclusion), partially renormalizing motility toward its control condition. Motility in such cases lay in between the control and hyperoxic exposure levels (e.g., effects of caffeine, GABA and MitoQ in HPAECs exposed to normobaric–hyperoxic conditions, middle panel on bottom row in Figures 1 and 2).

The comparisons of interest made with these results pertain directly to the effectiveness of any single drug's administration to mitigate the hyperoxia exposure effects on altering mitochondrial motility. Hence, the statistical differences we have assessed are those between any motility curve for a particular drug and environmental exposure and motility for the control condition of normobaric–normoxic exposure absent any drug. These comparisons are reliant on the magnitude of the geometric means of the mitochondrial displacements, which represent the central tendency of those displacements to follow a log-normal distribution. Magnitudes of the geometric means and those *p*-values determined for the associated statistical comparisons conducted are presented in Table 1 for experiments involving each of the different cell types studied [3, 17, 21, 22, 30, 42, 43, 46].

The small *p*-values appearing in Table 1 indicate that for all cases except one, and regardless of the directionality of shift of the motility induced by the presence of any drug, the motility distribution remains shifted significantly rightward from baseline. This indicates that mitochondrial motility is increased from control levels in each of the intracellular regions for all three cell types following every environmental exposure condition with each of the drugs present. The single exception case, highlighted in bold in Table 1, was found for motility in the intracellular peripheral region of A549 cells exposed to MitoQ and normobaric–normoxic conditions. This finding indicates that MitoQ had no effect on motility in this part of the cell absent any form of hyperoxic exposure. Overall these motility results indicate that all three of the drugs generally increase motility under normobaric–normoxic conditions, whereas their presence along with hyperoxic exposures has differential effects manifest either as further increased motility or partial normalization of motility toward baseline values. These effects are dependent on cell type, intracellular region, hyperoxic exposure type (normobaric versus hyperbaric), and drug type.

### 3.2. Drug Effects and Mitochondrial Inner Membrane Potential

**Figure 3:**
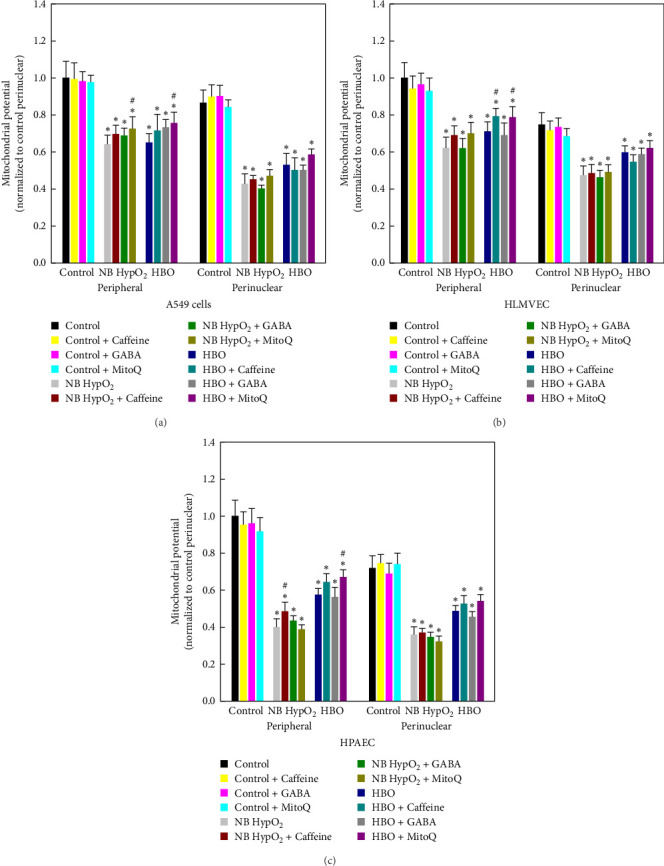
Hyperbaric/hyperoxic exposure, drug application, and mitochondrial inner membrane potential. tetramethylrhodamine methyl ester (TMRM)-based imaging results for inner membrane potential with A549 cells (a), HLMVECs (b), and HPAECs (c) under control (normobaric–normoxic) conditions, and after normobaric-hyperoxic (NB HypO_2_) and hyperbaric–hyperoxic (HBO) exposures and with each of the drugs added. Data appear as mean ± SD. *⁣*^*∗*^*p* < 0.05 in comparison to control in the indentical intracellular region for a particular cell type. #*p* < 0.05 compared to the hyperoxic exposure result in the same intracellular region for the same cell type and type of hyperoxic exposure.

TMRM image analysis performed to quantify mitochondrial inner membrane potential utilized cell images all presented at a singular image intensity. Results from this process of assessing the mean fluorescence intensity ratios are shown in Figure 3. These results were also used to perform statistical comparisons of effects of the drugs as well as effects of the different hyperoxic conditions imposed. As detailed in the Methods above, the inner membrane potential for every case has been normalized using the mean value determined in the perinuclear region for a particular cell type with control conditions [3, 22]. The results reflect that the hyperoxic exposures produced a significant reduction in mitochondrial inner membrane potential within the cell. Application of the drugs had only modest effects on the maintenance of the potential at control levels in only a few cases.

Specifically, the results in Figure 3 show that the drugs had no effect on the mitochondrial inner membrane potential under normobaric–normoxic conditions. Additionally, whether in the perinuclear or peripheral region of any cell type, and whether or not any drug was present, hyperoxic exposure at both normobaria and hyperbaria resulted in a significant loss of mitochondrial inner membrane potential compared to the normobaric–normoxic control level (*⁣*^*∗*^ in Figure 3). Moreover, only in a small number of individual cases (# in Figure 3; e.g., addition of MitoQ in A549 cells exposed to both types of hyperoxia, addition of caffeine and MitoQ for HLMVECs and HPAECs) was inner membrane potential partially preserved.. Of note, this was only evident within the intracellular peripheral region in those instances.

### 3.3. Drug Effects on Hyperoxia-Induced Alterations in Cellular Respiration

**Figure 4:**
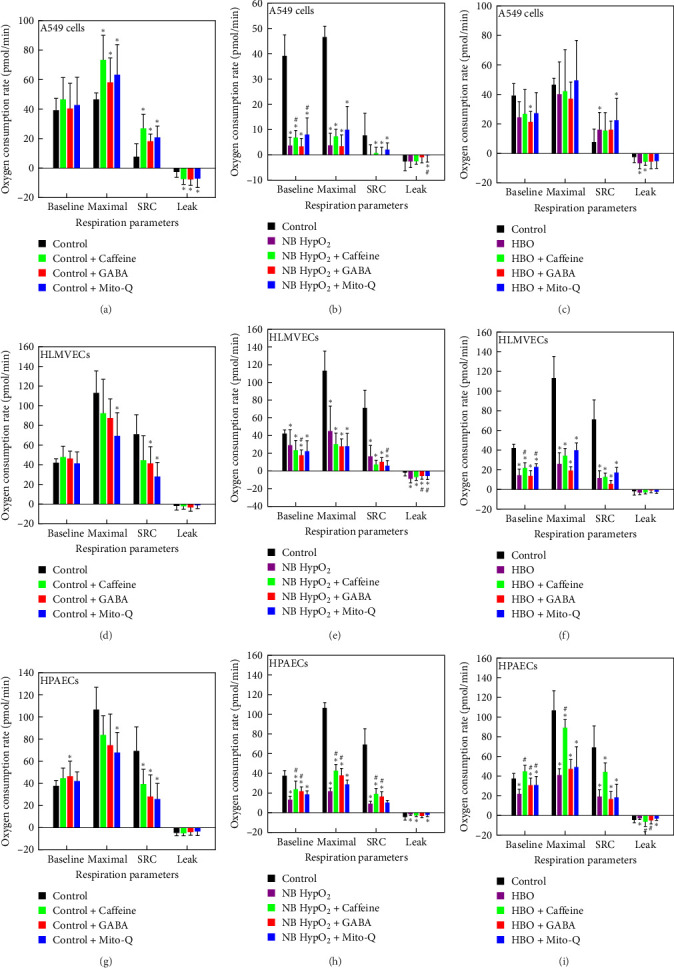
Drug effects on cellular respiration with hyperoxic exposures. Baseline respiration (baseline), maximal respiration (maximal), spare respiratory capacity (SRC), and proton leak (leak) with A549 cells (a, b, c), HLMVECs (d, e, f), and HPAECs (g, h, i) under normobaric–hyperoxic (control) conditions (a, d, g) and after normobaric–hyperoxic (NB HypO_2_, (b, e, h)) and hyperbaric–hyperoxic (HBO, (c, f, i)), with and without drugs present. Data appear as mean ± SD. *⁣*^*∗*^*p* < 0.05 in comparison to normobaric–normoxic controls for a particular cell type and respiration parameter. #*p* < 0.05 compared to the hyperoxic exposure result for the same respiration parameter without drug for the same cell type and hyperoxic exposure.

The results for cellular respiration measurements were characteristically similar to the findings for mitochondrial inner membrane potential. As shown in Figure 4, both types of hyperoxic exposure produced reductions in respiration parameters consistent with those reported previously [3, 22]. Under normobaric–normoxic conditions, for A549 cells each of the drugs increased maximal respiration, SRC, and proton leak, whereas the drugs had differential effects in HLMVECs to decrease maximal respiration (MitoQ) and SRC (GABA and MitoQ) and in HPAECs to increase baseline respiration (GABA), decrease maximal respiration (MitoQ) and decrease SRC (caffeine, GABA, and MitoQ). Following hyperbaric–hyperoxic exposure, nearly all respiration parameters were decreased from baseline in all three cell types with each of the drugs present. Addition of any of the drugs had only modest effects on the maintenance of any of the cellular respiration functions at control levels in only a few cases. Specifically, both caffeine and MitoQ partially preserved baseline respiration and MitoQ reduced proton leak in A549 cells exposed to normobaric–normoxia. GABA further reduced baseline respiration and MitoQ further reduced SRC, whereas GABA and MitoQ both decreased proton leak in HLMVECs exposed to normobaric normoxia. Caffeine and GABA partially maintained baseline and maximal respiration as well as SRC in HPAECs exposed to normobaric–normoxia. With hyperbaric–hyperoxic exposures, both caffeine and MitoQ maintained baseline respiration in HLMVECs, whereas all three drugs partially maintained baseline respiration in HPAECs. Additionally, caffeine partially maintained maximal respiration and SRC but increased proton leak, as did GABA, in HPAECs exposed to hyperbaric–hyperoxia.

### 3.4. Hyperbaric/Hyperoxic Exposure, Drug Application, and Partitioning of Intracellular Bioenergetics

**Figure 5:**
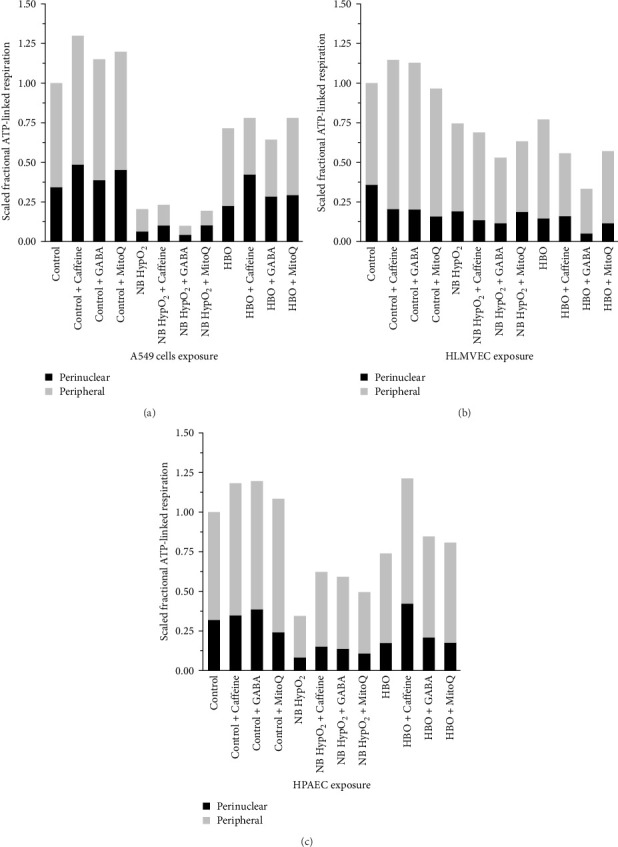
Drug effects on intracellular partitioning of bioenergetic capacity with hyperoxic exposures. Scaled fractional ATP-linked respiration in each of the intracellular subregions for A549 cells (a), HLMVECs (b), and HPAECs (c) for control (normobaric–normoxic) exposure and after normobaric–hyperoxic (NB HypO_2_) and hyperbaric–hyperoxic (HBO) exposures with and without caffeine, GABA, and MitoQ present.

Following the experimental approach used in earlier studies [3, 17, 21, 22, 43], we determined the bioenergetic capacity of each of the two distinct intracellular subregions, which are defined in the Methods section. The partitioning of the bioenergetic capacity between the intracellular perinuclear and peripheral spaces is determined by multiple factors. This includes the volume of mitochondria present within each space, which is dependent on mitochondrial size and number within each space. It also includes cellular respiration factors, especially the difference between baseline and proton leak respiration values, as well as the local inner membrane potential, which relates to the efficiency of the local mitochondria to produce ATP. All of these parameters were measured in the experiments as described previously. While independent measurement of respiration parameters cannot be performed directly within the two distinct intracellular subregions, our analysis methodology as noted above in the Methods section and as applied previously [3, 21, 22, 43] enables the determination of the scaled fractional ATP-linked respiration that occurred in each individual intracellular region under each experimental condition. The findings are shown in Figure 5, in which the portions of total ATP-linked respiration present within the two intracellular subregions appear in stacked bar form for every individual experimental condition. The control experiment level of total ATP-linked respiration for each cell type has been used to normalize these results.

As shown in Figure 5, the addition of all three drugs increased the full ATP-linked respiration from baseline in every cell type under normobaric–normoxic conditions, except for MitoQ in HLMVECs. With A549 cells, all three compounds also increased the perinuclear component of the total bioenergetic capacity from baseline, whereas only caffeine and GABA increased the perinuclear component from baseline in HPAECs. The perinuclear component of bioenergetic capacity was decreased from baseline by all three drugs in HLMVECs. Both types of hyperoxic exposures alone depressed whole ATP-linked respiration from baseline levels in every cell type, as previously reported [3, 22]. Compared to hyperoxic exposure alone, caffeine enhanced the perinuclear component of bioenergetic capacity in all three cell types undergoing both types of hyperoxic exposure, except in HLMVECs at normobaria. Additionally, GABA increased the perinuclear component in A549 cells for hyperbaric-hyperoxic exposure and in HPAECs for both normobaric- and hyperbaric–hyperoxic exposures compared to the hyperoxic conditions alone. MitoQ produced enhanced perinuclear ATP-linked respiration in A549 cells and HPAECs for both types of hyperoxic exposure, and in HLMVECs for normobaric–hyperoxic exposure, compared to the hyperoxic exposure alone.

**Figure 6:**
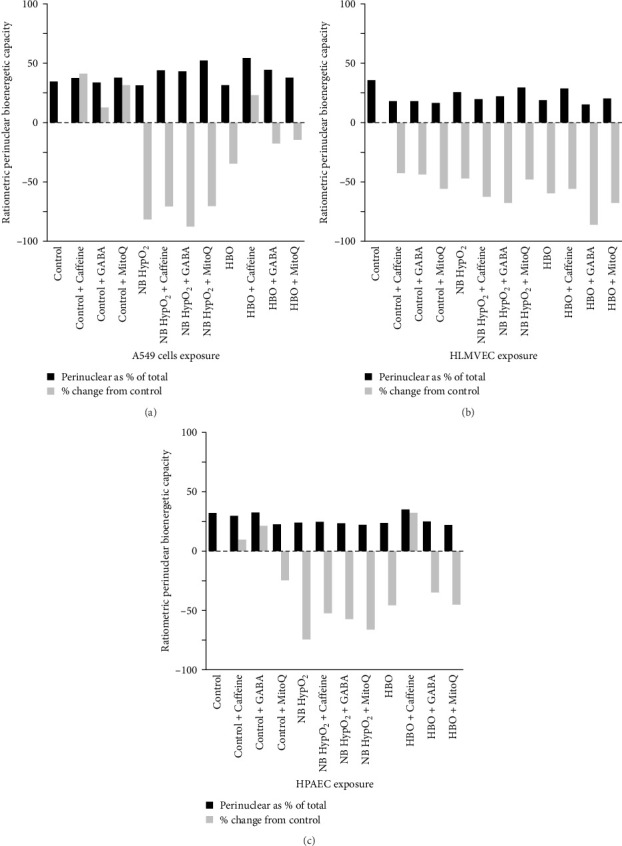
Drug effects on perinuclear bioenergetic capacity with hyperoxic exposures. ATP-linked respiration as a fraction of the whole amount at baseline with A549 cells (a), HLMVECs (b), and HPAECs (c) and after control (normobaric–normoxic), normobaric–hyperoxic (NB HypO_2_), and hyperbaric–hyperoxic (HBO) exposures with and without caffeine, GABA, and MitoQ present. Total percentage change in ATP-linked respiration within the perinuclear region from control (normobaric–normoxic exposure, no drug present) for each environmental exposure condition is shown as well.

By replotting the data appearing in Figure 5, the effectiveness of the various drugs to preserve the perinuclear bioenergetics in the various cell types at baseline (i.e., normobaric–normoxic) levels despite a hyperoxic exposure becomes evident.  Figure 6 presents both the fraction of whole cell ATP-linked respiration derived mitochondria residing in the perinuclear space as well as the percentage deviation from the baseline value for each of the cell types and experimental conditions imposed. This representation of the data makes it obvious that the perinuclear contribution, as a fractional component of the total cellular bioenergetic capacity, appears to be maintained within a fairly small range. However, especially in the cases of hyperoxic exposures, there is primarily a large percentage decrease in the perinuclear available ATP-derived bioenergy compared to control for every cell type. An important distinction is that caffeine produced a higher (larger positive percentage) difference from control with both A549 cells and HPAECs exposed to hyperbaric–hyperoxia.

### 3.5. Redistribution of Mitochondrial Volume

**Figure 7:**
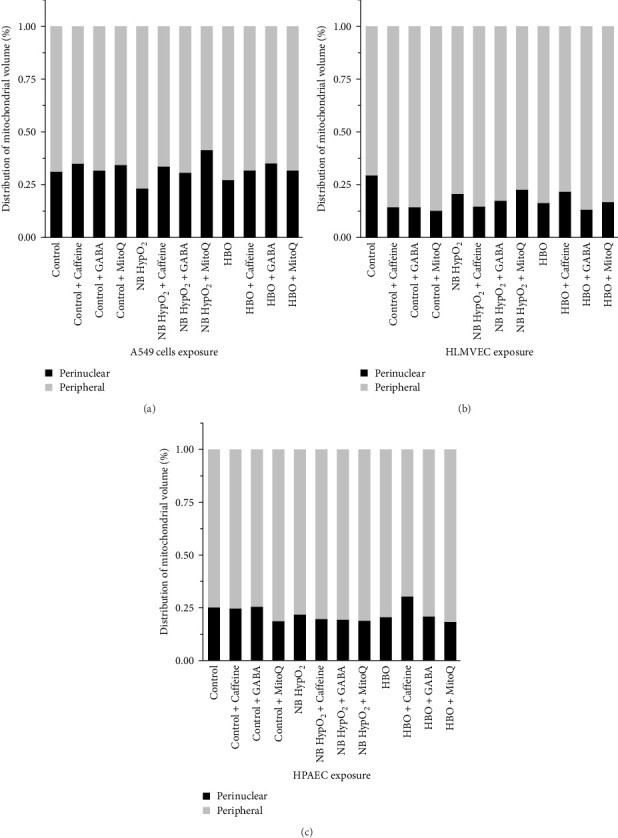
Drug and hyperoxia effects on perinuclear mitochondrial volume. Stacked bar graphs of the fractional distribution of mitochondrial volume located within each of the intracellular regions for A549 cells (a), HLMVECs (b), and HPAECs (c) under control (normobaric–normoxic) conditions and after normobaric–hyperoxic (NB HypO_2_) and hyperbaric–hyperoxic (HBO) exposures for caffeine, GABA, and MitoQ being absent or present.

In order to reconcile overall partitioning of intracellular bioenergetic capacity with preservation of its perinuclear component for the various cell types and different experimental conditions as indicated in Figures 5 and 6, we note that differences in cellular respiration levels as shown in Figure 4 and mitochondrial inner membrane potential (Figure 3) only partially account for these results. Additional insights can be drawn from the effects of the various drugs to promote redistribution of the mitochondrial volume within the cells. As shown in Figure 7, the fraction of the mitochondrial volume that is present in the intracellular perinuclear region is dependent on both the environmental exposure condition as well as the presence of any of the drug. One consistent feature of the plots depicted in Figure 7 is the effect of caffeine to increase the volume of mitochondria present in the perinuclear region from control in every cell type for hyperbaric-hyperoxic conditions. While otherwise the effects of the drugs are variable across the cell types and environmental exposure conditions (e.g., MitoQ enhancement of perinuclear mitochondrial volume in A549 cells and HLMVECs exposed to normobaric–hyperoxia), this single finding regarding caffeine helps to clarify the results reported above for Figure 6 in interpreting its influence on ATP availability to the nucleus in the face of hyperbaric–hyperoxia.

### 3.6. Mitochondrial ETC Protein Levels

**Figure 8:**
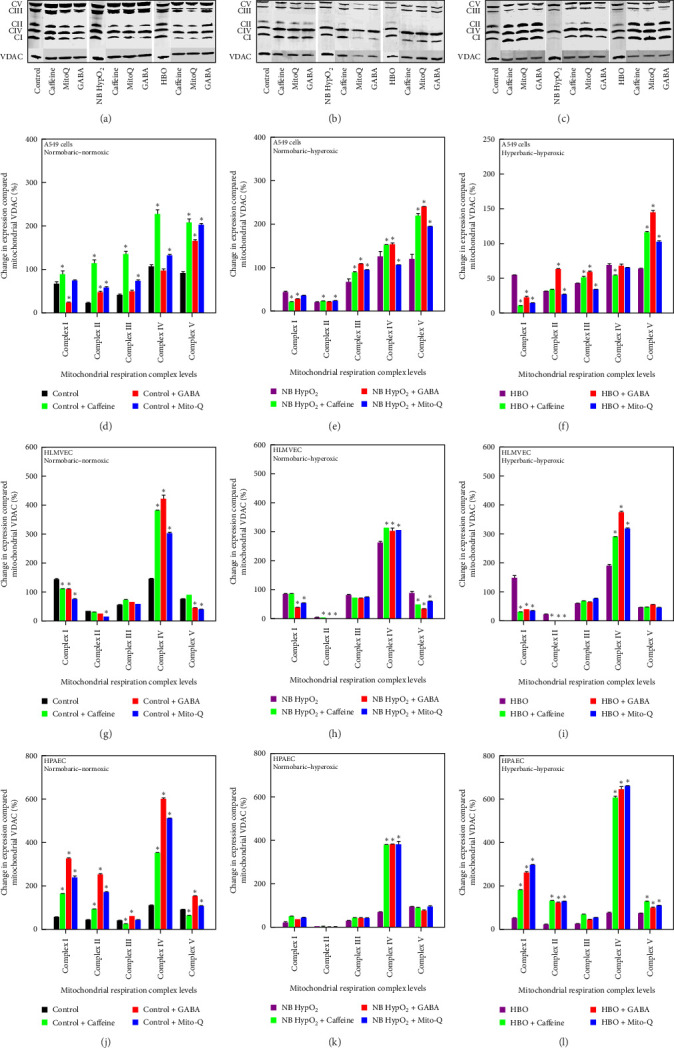
Western blots and quantitation of ETC protein levels. Western blots for respiration complexes I–V and VDAC for A549 cells (a), HLMVECs (b), and HPAECs (c) at baseline and following normobaric–hyperoxic and hyperbaric–hyperoxic exposures. densitometry results for ETC protein expression levels in A549 cells (d, e, f), HLMVECs (g, h, i), and HPAECs (j, k, l) under normobaric–normoxic (control) conditions and after normobaric–hyperoxic (NB HypO_2_) and hyperbaric–hyperoxic (HBO) exposures. Results are normalized by VDAC levels. Data (*n* = 3) are shown as mean ± SD. *⁣*^*∗*^*p* < 0.05 in comparison to the drug-free state for each environmental condition.

Images of Western blots for ETC protein and VDAC levels present in every cell type studied under each of the environmental exposure conditions with and without drugs applied are demonstrated in Figure 8 (top). The lower portion of Figure 8 shows the quantification of these protein levels for each cell type under each environmental condition with and without the drugs applied as determined via densitometric analysis. All results have been normalized to the VDAC levels as defined above in the Methods section. Previous work [3, 22] also reported that levels of an additional protein, β-tubulin, were not altered by hyperoxic exposure, indicating that changes in mitochondrial motility are not the result of alterations in tubulin levels affecting any interplay between this protein and molecular motor structures involved in mitochondrial motion.

Each of the drugs produced mixed effects on individual complex levels, depending on cell type and environmental exposure conditions. This is evidenced by the drug-associated decreases occurring in Complex I and Complex II levels in A549 cells, especially with hyperoxic exposures, and in Complex I, Complex II, and Complex V levels in HLMVECs, which were present to a large degree for both normoxic and hyperoxic conditions. However, among the effects of the drugs to elevate complex levels, there were significant increases in Complex IV levels in all three cell types for all three of the drugs studied occurring with both types of hyperoxia exposure. The single exception to this occurred for A549 cells undergoing hyperbaric-hyperoxic conditions. In that case, caffeine use reduced the Complex IV level.

## 4. Discussion

Oxygen is an essential molecule for the production of energy and maintenance of health in mammalian life. However, large elevations of breathable oxygen levels—hyperoxia—can lead to severely detrimental and damaging effects on cells and tissues. The lung is one organ that is particularly vulnerable to insult from hyperoxia [49]. Pulmonary oxygen toxicity has been a known pathophysiological condition for more than a century [50]. A key feature to the onset of pulmonary toxicity is that it develops on different timescales depending on the ambient atmospheric pressure. Under normobaric conditions, it arises over the course of 2–3 days, especially if the oxygen concentration is well above 50% [3, 51–57]. Under hyperbaric conditions with close to 100% oxygen being present, evidence of oxygen toxicity can appear in as little as an hour at 4.8 ATA [22], and it has been shown to be fulminant in animal models in 6–10 h using lower pressure hyperbaric exposures [15]. These two different pressure conditions are part of a time/oxygen content/pressure relationship that produces pulmonary oxygen toxicity, and they are representative of two distinct cohorts who are prone to develop this illness: hospitalized patients who are intubated and mechanically ventilated in the ICU setting, and underwater divers. ICU patients can have a variety of conditions (e.g., sepsis, heart failure, acute lung injury, and severe COPD) leading to tissue hypoxemia that requires significant oxygen supplementation in a one-atmosphere environment. Thus, the lung is subjected to conditions that lead to oxygen toxicity over longer time because prolonged use of a high oxygen concentration is the only available clinical option with no alternative therapy being available. Within the sport, commercial, and military diving communities, individuals undergo exposure to oxygen toxicity occurring in shorter timeframes due to the increased external pressure experienced with submergence. These two types of exposure, normobaric- and hyperbaric–hyperoxic conditions, have been demonstrated to produce forms of pulmonary oxygen toxicity that have a number of similar features, yet also maintain some differences. As noted in the Introduction section, pulmonary oxygen toxicity associated with normobaric exposures includes diffuse damage in pulmonary capillary endothelial cells and also alveolar epithelial cells. The associated pathophysiology includes inflammatory cell infiltration, intra-alveolar and interstitial edema, and significant decrements in gas exchange [9–12]. Pulmonary oxygen toxicity caused by hyperbaric–hyperoxic exposure features similar microscopic and gross pathological tissue changes, but it exhibits less direct inflammatory-related injury and more noninflammatory-related injury than occurs in its normobaric counterpart [15].

Our earlier pulmonary cell-based experiments with normobaric–hyperoxic [3] and hyperbaric–hyperoxic [22] exposures have borne out that many of the mitochondrial bioenergetics and dynamics characteristics of oxygen toxicity resulting from these different conditions are similar, but not identical. The molecular, cellular, tissue, and organ responses to hyperoxia in vitro and in vivo generally have been ascribed to increased production of reactive oxygen species (ROSs) and the oxidative stress and injury that incurs. In particular, mitochondrial ROS become elevated in oxygen toxicity [7], creating a signaling axis that involves the mitochondria as well as the cell nucleus. This axis contributes to multiple cellular functional responses including inflammatory responses and apoptotic responses leading to cell death, especially in endothelial cells [58]. These responses, as well as the emergence of changes in mitochondrial locomotion (i.e., motility) and respiration can indicate that inadequate bioenergy is available to cells to maintain cellular homeostasis. This may also be reflected in oxygen toxicity-related alterations in the proteome [59] and gene expression [60, 61] and other elements of metabolic activity [62–64] susceptible to impairment by high oxygen environments.

While ROS are implicated in the physiological disturbances that occur in oxygen toxicity, to date there is no reliably effective drug, including any antioxidant, has been shown to alter development of pulmonary oxygen toxicity in humans. Instead, clinical approaches to avoidance of pulmonary oxygen toxicity at normobaria rely on employing as low an oxygen concentration as possible when using supplemental oxygen, while diving approaches to avoidance of oxygen toxicity at hyperbaria rely on keeping submergence time as short as possible during oxygen dives. Neither of these approaches has changed in decades. This fact underlies our methodology for producing oxygen toxicity in pulmonary cells in vitro under both normobaric and hyperbaric conditions. These distinct environmental exposure models provide platforms for distinguishing therapeutic options that might be useful in treating one or both of the subforms of pulmonary oxygen toxicity that are acknowledged to affect individuals [15]. Moreover, studying therapeutics in a framework of observable mitochondrial molecular dysfunction that manifests the presence of oxygen toxicity at a subcellular level provides deeper insights into strategies to prevent toxicity-caused disturbances in cellular respiration [65], mitochondrial dynamics [42], or alterations in respiration function as well as mitochondrial dynamics [46]. Our application of caffeine [66–69], GABA [12, 70], and MitoQ [71, 72] in these experiments is based on their demonstrated utility in published studies of oxygen toxicity or mitochondrial dysfunction. As such, these are the first studies to examine their effects on mitochondrial bioenergetic and dynamic markers of pulmonary oxygen toxicity.

The various mitochondrial assessments which we have performed of the three potential therapeutics' effects on oxygen toxicity illustrate overall self-consistency for each of the drugs studied. This is illustrated in Figures 1–7 and Table 1 in terms of their influences on mitochondrial respiration, morphology, motility, maintenance of inner membrane potential, and redistribution of bioenergy within the cell, including its repartitioning between the peripheral and perinuclear spaces. Under baseline conditions in the absence of hyperoxia, all three drugs generally increase mitochondrial motility and cause, at most, very modest changes in baseline and/or maximal respiration in all three cell types examined. They do not cause any change in mitochondrial intermembrane potential. All three drugs result in an increase in the perinuclear mitochondrial volume and increased fraction of perinuclear ATP-linked respiration in A549 cells as well as a decrease in the perinuclear mitochondrial volume and decreased fraction of perinuclear ATP-linked respiration in HLMVECs. Caffeine and GABA appear to increase perinuclear ATP-linked respiration without changing perinuclear mitochondrial volume in HLMVECs, while MitoQ reduced both parameters in these cells.

With hyperoxia exposure the effects of the drugs on alterations in these features of mitochondrial bioenergetics and dynamics are shown to be particularly sensitive both to the means by which oxygen toxicity is induced in the cells (i.e., normobaric- or hyperbaric–hyperoxic exposure), and the particular cell type being studied. For instance, Figure 5 shows that the scaled fractional ATP-linked respiration is more depressed and less responsive to any of the drugs in both A549 cells and HPAECs than in HLMVECs when undergoing normobaric–hyperoxic exposure compared to hyperbaric-hyperoxic exposure. This is evident as well in Figure 6, which further delineates that caffeine is the only one of the three drugs that elicited a net increase in the perinuclear component of bioenergy in a hyperoxic condition, as seen for A549 cells and HPAECs undergoing hyperbaric–hyperoxic exposure. We might have thought all three drugs could yield such positive effects based on changes in perinuclear bioenergetic capacity found under baseline normobaric–normoxic conditions (Figure 6). However, when the cells were challenged by hyperoxia, only caffeine as noted above had any positive effect to preserve or increase the nucleus' energy availability. As we further demonstrated, this energy availability was facilitated by a caffeine-associated shift in mitochondrial volume from the cell periphery to the perinuclear region in these cells (Figure 7). While such a shift also occurred with MitoQ and GABA in A549 cells under both types of hyperoxic exposure and with caffeine in HLMVECs exposed to hyperbaric–hyperoxia, the effect was insufficient to overcome the overall depression in total energy production brought about by these environmental hyperoxic conditions.

This limitation of bioenergy availability is also reflected in the mitochondrial motility results showing significant increases in motility as a result of impaired energy availability driving increased substrate seeking activity by mitochondria through greater sampling of their local environment for metabolic material (Figures 1 and 2). The results in Figure 8 indicate that increased abundance of certain ETC proteins, especially Complex IV as additional ATP-processing machinery with each of the drugs added, could also facilitate bioenergy generation as an additional compensatory mechanism. We note that it is not known how any of the respiration complex subunits respond to hyperoxic and/or hyperbaric exposure or to the drugs themselves. Thus, we realize that detail regarding specific changes in the abundance of any of the respiratory complexes presented in Figure 8 is not conclusive for any single subunit of any complex assessed by the Western blotting we have performed. This could influence other effects of metabolic disturbances occurring within the mitochondrial OXPHOS system, as suggested elsewhere [73]. However, we are confident that our methods are reasonable and that the results we have obtained accurately indicate the effects of the drugs to mitigate oxygen toxicity.

## 5. Conclusion

The present investigations demonstrate that caffeine, more so than GABA and MitoQ, preserves mitochondrial bioenergetics and dynamics following pulmonary cell exposure to hyperbaric–hyperoxia. The underlying mechanisms include the promotion of redistribution of mitochondrial volume within the cell interior and an increased abundance of ETC proteins. Further study may identify specific mitochondrial-targeted therapeutics having clinical potential for application in the treatment or prevention of normobaric and/or hyperbaric forms of pulmonary oxygen toxicity.

## Figures and Tables

**Table 1:** Geometric mean of the net distances traveled by mitochondria within the two intracellular regions in A549, HLMVEC, and HPAECs and associated *p*-values for group comparisons.

Cell type	Exposure conditions		Cell perinuclear region				Cell periphereal region			
			No drug	Caffeine	GABA	Mito-Q	No drug	Caffeine	GABA	Mito-Q
A549 cells	Normobaric–normoxia	Geometric mean net distance (nm)	65.34	81.18	80.55	71.76	76.42	88.9	92.82	77.98
		*p*-Value	N/A	<1E−15	1.48E−12	<1E−15	N/A	1.37E−13	1.06E−10	**1.11E−01**
	Normobaric–hyperoxia	Geometric mean net distance (nm)	75.4	77.95	84.79	70.45	91.32	96.24	99.21	88.04
		*p*-Value	8.79E−10	<1E−15	<1E−15	<1E−15	7.63E−09	8.99E−09	2.27E−10	7.62E−03
	Hyperbaric–hyperoxia	Geometric mean net distance (nm)	53.59	96.52	82.54	84.6	62.67	112.78	85.45	100.22
		*p*-Value	<1E−15	<1E−15	<1E−15	<1E−15	<1E−15	<1E−15	2.18E−09	2.65E−06

HLMVEC	Normobaric–normoxia	Geometric mean net distance (nm)	72.46	96.52	95.49	89.51	88.2	78.86	83.85	74.48
		*p*-Value	N/A	<1E−15	4.33E−12	1.37E−14	N/A	4.09E−06	2.03E−06	3.11E−09
	Normobaric–hyperoxia	Geometric mean net distance (nm)	98.99	97.4	98.46	83.14	82.07	78.29	94.38	82
		*p*-Value	<1E−15	3.49E−09	<1E−15	2.04E−04	2.30E−02	2.53E−07	1.29E−07	3.24E−03
	Hyperbaric–hyperoxia	Geometric mean net distance (nm)	100.5	98.42	86.38	106.85	105.31	97.51	92.71	94.75
		*p*-Value	<1E−15	<1E−15	1.71E−09	2.70E−14	<1E−15	1.24E−11	6.78E−06	4.06E−07

HPAECs	Normobaric–normoxia	Geometric mean net distance (nm)	60.09	79.7	81.03	71.43	54.53	84	81.48	81.89
		*p*-Value	N/A	<1E−15	6.18E−13	<1E−15	N/A	1.33E−15	7.12E−06	5.26E−10
	Normobaric–hyperoxia	Geometric mean net distance (nm)	92.65	82.19	71.67	78.77	96.97	79.29	81.91	80.53
		*p*-Value	<1E−15	<1E−15	<1E−15	<1E−15	<1E−15	<1E−15	<1E−15	<1E−15
	Hyperbaric–hyperoxia	Geometric mean net distance (nm)	94.27	101.07	113.05	98.6	101.15	106.41	109.43	98.26
		*p*-Value	<1E−15	<1E−15	<1E−15	<1E−15	<1E−15	<1E−15	<1E−15	<1E−15

*Note:* Comparisons reported are for results of a particular experimental condition (exposure ± drug) and the results obtained for normobaric–normoxic exposure without any drug being present.

## Data Availability

The data that support the findings of this study are available from the corresponding author upon reasonable request.
